# Crystal structure of the coordination polymer [Fe^III^_2_{Pt^II^(CN)_4_}_3_]

**DOI:** 10.1107/S2056989014026188

**Published:** 2015-01-01

**Authors:** Maksym Seredyuk, M. Carmen Muñoz, José A. Real, Turganbay S. Iskenderov

**Affiliations:** a National Taras Shevchenko University, Department of Chemistry, Volodymyrska str. 64, 01601 Kyiv, Ukraine; b Departamento de Fisica Aplicada, Universitat Politecnica de Valencia, Camino de Vera s/n, 46022 Valencia, Spain; c Institut de Ciencia Molecular (ICMol), Departament de Quimica Inorganica, Universitat de Valencia, C/Catedratico José Beltran Martinez, 2, 46980, Paterna, Valencia, Spain

**Keywords:** crystal structure, polycyanidometalate, spin-crossover

## Abstract

The title complex, poly[dodeca-μ-cyanido-diiron(III)triplat­inum(II)], [Fe^III^_2_{Pt^II^(CN)_4_}_3_], has a three-dimensional polymeric structure. It is built-up from square-planar [Pt^II^(CN)_4_]^2−^ anions (point group symmetry 2/*m*) bridging cationic [Fe^III^Pt^II^(CN)_4_]^+^_∞_ layers extending in the *bc* plane. The Fe^II^ atoms of the layers are located on inversion centres and exhibit an octa­hedral coordination sphere defined by six N atoms of cyanide ligands, while the Pt^II^ atoms are located on twofold rotation axes and are surrounded by four C atoms of the cyanide ligands in a square-planar coordination. The geometrical preferences of the two cations for octa­hedral and square-planar coordination, respectively, lead to a corrugated organisation of the layers. The distance between neighbouring [Fe^III^Pt^II^(CN)_4_]^+^_∞_ layers corresponds to the length *a*/2 = 8.0070 (3) Å, and the separation between two neighbouring Pt^II^ atoms of the bridging [Pt^II^(CN)_4_]^2−^ groups corresponds to the length of the *c* axis [7.5720 (2) Å]. The structure is porous with accessible voids of 390 Å^3^ per unit cell.

## Related literature   

Coordination compounds have inter­esting properties in catal­ysis (Kanderal *et al.*, 2005 ▸; Penkova *et al.*, 2009 ▸) or as photoactive materials (Yan *et al.*, 2012 ▸). Magnetically active polycyanidometallate network complexes of Fe^II^ [Fe^II^ *L*_2_{*M* ^I^(CN)_2_}_2_] or [Fe^II^ *L*_2_{*M* ^II^(CN)_4_}] (*M* ^I^ = Ag, Au; *M* ^II^ = Ni, Pd, Pt; *L* = *N*-heterocyclic ligand) have been studied because they show versatile polymeric structures (Piñeiro-López *et al.* 2014 ▸; Seredyuk *et al.*, 2007 ▸, 2009 ▸), spin transition (Muñoz & Real, 2013 ▸) and functionalities such as sorption–desorption of organic and inorganic mol­ecules (Muñoz & Real, 2013 ▸) or reversible chemosorption (Arcís-Castillo *et al.*, 2013 ▸).

## Experimental   

### Crystal data  

[Fe_2_Pt_3_(CN)_12_]
*M* *_r_* = 1009.18
Monoclinic, 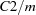

*a* = 16.0140 (5) Å
*b* = 13.8250 (5) Å
*c* = 7.5720 (2) Å
β = 102.946 (2)°
*V* = 1633.78 (9) Å^3^
*Z* = 2
Mo *K*α radiation
μ = 13.68 mm^−1^
*T* = 293 K
0.04 × 0.04 × 0.02 mm

### Data collection  

Oxford Diffraction Gemini S Ultra diffractometer
Absorption correction: multi-scan (Blessing, 1995 ▸) *T*_min_ = 0.611, *T*_max_ = 0.772
3358 measured reflections
1909 independent reflections
1568 reflections with *I* > 2σ(*I*)
*R*_int_ = 0.038

### Refinement  

*R*[*F* ^2^ > 2σ(*F* ^2^)] = 0.038
*wR*(*F* ^2^) = 0.106
*S* = 0.97
1909 reflections
71 parameters
Δρ_max_ = 1.25 e Å^−3^
Δρ_min_ = −1.33 e Å^−3^

### 

Data collection: *COLLECT* (Nonius, 1999 ▸); cell refinement: *SCALEPACK* (Otwinowski & Minor, 1997 ▸); data reduction: *DENZO* (Otwinowski & Minor, 1997 ▸) and *SCALEPACK*; program(s) used to solve structure: *SHELXS97* (Sheldrick, 2008 ▸); program(s) used to refine structure: *SHELXL97* (Sheldrick, 2008 ▸); molecular graphics: *DIAMOND* (Brandenburg, 1999 ▸); software used to prepare material for publication: *WinGX* (Farrugia, 2012 ▸).

## Supplementary Material

Crystal structure: contains datablock(s) I. DOI: 10.1107/S2056989014026188/wm5094sup1.cif

Structure factors: contains datablock(s) I. DOI: 10.1107/S2056989014026188/wm5094Isup2.hkl

**Fig. 1.**
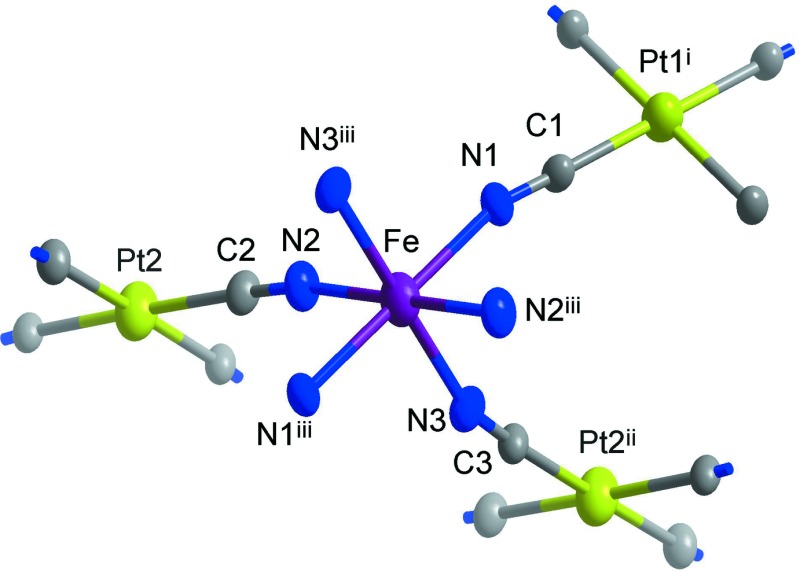
Displacement ellipsoid plot (30% probability level) of the principal building units of the structure of the title compound. [Symmetry codes: (i) 1/2 + x, 1/2 + y, 1 + z; (ii) 0.5 – x, 1/2 + y, 1 – z, (iii) x, 1 – y, 1 + z.]

**Fig. 2.**
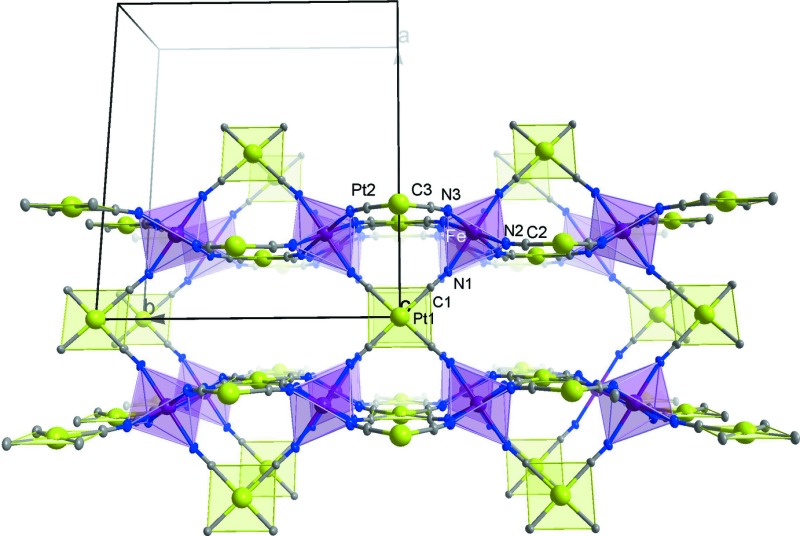
A fragment of three-dimentional coordination polymer of the title compound in a perspective view along c. Polyhedra correspond to FeN6 and PtC4 chromophores.

Supporting information file. DOI: 10.1107/S2056989014026188/wm5094Isup3.cdx

x y z x y z x y z . DOI: 10.1107/S2056989014026188/wm5094fig1.tif
Displacement ellipsoid plot (30% probability level) of the principal building units of the structure of the title compound. [Symmetry codes: (i) 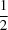 + *x*, 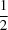 + *y*, 1 + *z*; (ii) 0.5 – *x*, 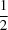 + *y*, 1 – *z*, (iii) *x*, 1 – *y*, 1 + *z*.]

c 6 4 . DOI: 10.1107/S2056989014026188/wm5094fig2.tif
A fragment of three-dimentional coordination polymer of the title compound in a perspective view along *c*. Polyhedra correspond to FeN_6_ and PtC_4_ chromophores.

CCDC reference: 1036669

Additional supporting information:  crystallographic information; 3D view; checkCIF report

## supplementary crystallographic information

### S1. Synthesis and crystallization

Single crystals of the title compound were grown using a slow diffusion technique. During the reaction time a side product had formed serendipitously due to oxidation of the initial Fe^II^ salt. One side of a multi-arm shaped vessel contained (NH_4_)_2_Fe(SO_4_)_2_·6H_2_O (20 mg, 51 mmol) dissolved in water (0.5 mL). The second arm contained K_2_[Pt(CN)_4_]·3H_2_O (22 mg, 51 mmol) in water (0.5 ml). The vessel was filled with a water/methanol (1:1) solution. Square shaped orange crystals suitable for single crystal X-ray analysis were obtained after several weeks.

### S2. Refinement

The highest and lowest remaining electron density are located 3.66 and 0.83 Å, respectively, from the Pt atom. The highest electron densities are connected with positions in the voids of the framework. However, modelling of the electron density *e.g.* under consideration of disordered (partially occupied) water molecules lead to implausible models.

### Crystal data

**Table:**

[Fe_2_Pt_3_(CN)_12_]	*F*(000) = 884
*M**_r_* = 1009.18	*D*_x_ = 2.051 Mg m^−^^3^
Monoclinic, *C*2/*m*	Mo *K*α radiation, λ = 0.71073 Å
Hall symbol: -C 2y	Cell parameters from 200 reflections
*a* = 16.0140 (5) Å	θ = 12–20°
*b* = 13.8250 (5) Å	µ = 13.68 mm^−^^1^
*c* = 7.5720 (2) Å	*T* = 293 K
β = 102.946 (2)°	Prismatic, orange
*V* = 1633.78 (9) Å^3^	0.04 × 0.04 × 0.02 mm
*Z* = 2	

### Data collection

**Table:**

Oxford Diffraction Gemini S Ultra diffractometer	1909 independent reflections
Radiation source: fine-focus sealed tube	1568 reflections with *I* > 2σ(*I*)
Graphite monochromator	*R*_int_ = 0.038
ω scans	θ_max_ = 27.5°, θ_min_ = 3.0°
Absorption correction: multi-scan (Blessing, 1995)	*h* = −20→20
*T*_min_ = 0.611, *T*_max_ = 0.772	*k* = −17→16
3358 measured reflections	*l* = −9→9

### Refinement

**Table:**

Refinement on *F*^2^	0 constraints
Least-squares matrix: full	Primary atom site location: structure-invariant direct methods
*R*[*F*^2^ > 2σ(*F*^2^)] = 0.038	Secondary atom site location: difference Fourier map
*wR*(*F*^2^) = 0.106	*w* = 1/[σ^2^(*F*_o_^2^) + (0.0615*P*)^2^ + 15.455*P*] where *P* = (*F*_o_^2^ + 2*F*_c_^2^)/3
*S* = 0.97	(Δ/σ)_max_ < 0.001
1909 reflections	Δρ_max_ = 1.25 e Å^−^^3^
71 parameters	Δρ_min_ = −1.33 e Å^−^^3^
0 restraints	

### Special details

**Table:**

Geometry. All e.s.d.'s (except the e.s.d. in the dihedral angle between two l.s. planes) are estimated using the full covariance matrix. The cell e.s.d.'s are taken into account individually in the estimation of e.s.d.'s in distances, angles and torsion angles; correlations between e.s.d.'s in cell parameters are only used when they are defined by crystal symmetry. An approximate (isotropic) treatment of cell e.s.d.'s is used for estimating e.s.d.'s involving l.s. planes.
Refinement. Refinement of *F*^2^ against ALL reflections. The weighted *R*-factor *wR* and goodness of fit *S* are based on *F*^2^, conventional *R*-factors *R* are based on *F*, with *F* set to zero for negative *F*^2^. The threshold expression of *F*^2^ > σ(*F*^2^) is used only for calculating *R*-factors(gt) *etc*. and is not relevant to the choice of reflections for refinement. *R*-factors based on *F*^2^ are statistically about twice as large as those based on *F*, and *R*-factors based on ALL data will be even larger.

### Fractional atomic coordinates and isotropic or equivalent isotropic displacement parameters (Å^2^)

**Table:**

	*x*	*y*	*z*	*U*_iso_*/*U*_eq_	
Pt1	0.0000	0.0000	0.0000	0.02376 (17)	
Pt2	0.19452 (3)	0.5000	0.47749 (5)	0.02524 (16)	
Fe	0.2500	0.2500	0.0000	0.0215 (3)	
N1	0.1335 (5)	0.1622 (5)	−0.0284 (10)	0.0368 (17)	
N2	0.2081 (6)	0.3449 (5)	0.1843 (10)	0.0400 (18)	
N3	0.3039 (6)	0.1577 (5)	0.2273 (10)	0.0385 (17)	
C1	0.0859 (5)	0.1023 (6)	−0.0190 (12)	0.0310 (17)	
C2	0.2001 (6)	0.4002 (6)	0.2915 (11)	0.0335 (19)	
C3	0.3072 (6)	0.1012 (6)	0.3373 (10)	0.0312 (18)	

### Atomic displacement parameters (Å^2^)

**Table:**

	*U*^11^	*U*^22^	*U*^33^	*U*^12^	*U*^13^	*U*^23^
Pt1	0.0208 (3)	0.0167 (3)	0.0343 (3)	0.000	0.0073 (2)	0.000
Pt2	0.0389 (3)	0.0182 (2)	0.0195 (2)	0.000	0.00824 (18)	0.000
Fe	0.0294 (8)	0.0165 (7)	0.0199 (7)	−0.0040 (6)	0.0083 (6)	−0.0004 (5)
N1	0.041 (4)	0.026 (4)	0.042 (4)	−0.009 (3)	0.008 (4)	−0.002 (3)
N2	0.056 (5)	0.030 (4)	0.038 (4)	−0.004 (4)	0.017 (4)	−0.006 (3)
N3	0.053 (5)	0.026 (4)	0.037 (4)	0.002 (4)	0.011 (4)	0.006 (3)
C1	0.028 (4)	0.023 (4)	0.043 (4)	0.000 (3)	0.011 (4)	0.004 (3)
C2	0.050 (6)	0.026 (4)	0.026 (4)	0.003 (4)	0.012 (4)	−0.001 (3)
C3	0.045 (5)	0.021 (4)	0.025 (4)	−0.001 (4)	0.004 (4)	0.000 (3)

### Geometric parameters (Å, º)

**Table:**

Pt1—C1	2.000 (8)	Fe—N2	2.130 (7)
Pt1—C1^i^	2.000 (8)	Fe—N3^vii^	2.161 (7)
Pt1—C1^ii^	2.000 (8)	Fe—N3	2.161 (7)
Pt1—C1^iii^	2.000 (8)	Fe—N1^vii^	2.195 (7)
Pt2—C3^iv^	1.986 (8)	Fe—N1	2.195 (7)
Pt2—C3^v^	1.986 (8)	N1—C1	1.139 (10)
Pt2—C2	1.988 (8)	N2—C2	1.143 (11)
Pt2—C2^vi^	1.988 (8)	N3—C3	1.134 (10)
Fe—N2^vii^	2.130 (7)	C3—Pt2^v^	1.986 (8)
			
C1—Pt1—C1^i^	90.0 (5)	N3^vii^—Fe—N3	180.0 (3)
C1—Pt1—C1^ii^	180.0 (6)	N2^vii^—Fe—N1^vii^	91.1 (3)
C1^i^—Pt1—C1^ii^	90.0 (5)	N2—Fe—N1^vii^	88.9 (3)
C1—Pt1—C1^iii^	90.0 (5)	N3^vii^—Fe—N1^vii^	86.0 (3)
C1^i^—Pt1—C1^iii^	180.0 (6)	N3—Fe—N1^vii^	94.0 (3)
C1^ii^—Pt1—C1^iii^	90.0 (5)	N2^vii^—Fe—N1	88.9 (3)
C3^iv^—Pt2—C3^v^	89.6 (4)	N2—Fe—N1	91.1 (3)
C3^iv^—Pt2—C2	178.1 (4)	N3^vii^—Fe—N1	94.0 (3)
C3^v^—Pt2—C2	91.2 (3)	N3—Fe—N1	86.0 (3)
C3^iv^—Pt2—C2^vi^	91.2 (3)	N1^vii^—Fe—N1	180.0 (2)
C3^v^—Pt2—C2^vi^	178.1 (4)	C1—N1—Fe	164.2 (7)
C2—Pt2—C2^vi^	87.9 (5)	C2—N2—Fe	168.3 (8)
N2^vii^—Fe—N2	180.0 (5)	C3—N3—Fe	159.4 (8)
N2^vii^—Fe—N3^vii^	88.3 (3)	N1—C1—Pt1	178.3 (7)
N2—Fe—N3^vii^	91.7 (3)	N2—C2—Pt2	175.9 (9)
N2^vii^—Fe—N3	91.7 (3)	N3—C3—Pt2^v^	176.4 (8)
N2—Fe—N3	88.3 (3)		

Symmetry codes: (i) *x*, −*y*, *z*; (ii) −*x*, −*y*, −*z*; (iii) −*x*, *y*, −*z*; (iv) −*x*+1/2, *y*+1/2, −*z*+1; (v) −*x*+1/2, −*y*+1/2, −*z*+1; (vi) *x*, −*y*+1, *z*; (vii) −*x*+1/2, −*y*+1/2, −*z*.
