# Supplementary figures and images for: Crystal structure of the coordination polymer [FeIII 2{PtII(CN)4}3]
Source: Acta Crystallogr E Crystallogr Commun. 2015 Jan 1;71(Pt 1):i1–2. doi: 10.1107/S2056989014026188 (PMC4331858; doi:10.1107/S2056989014026188)

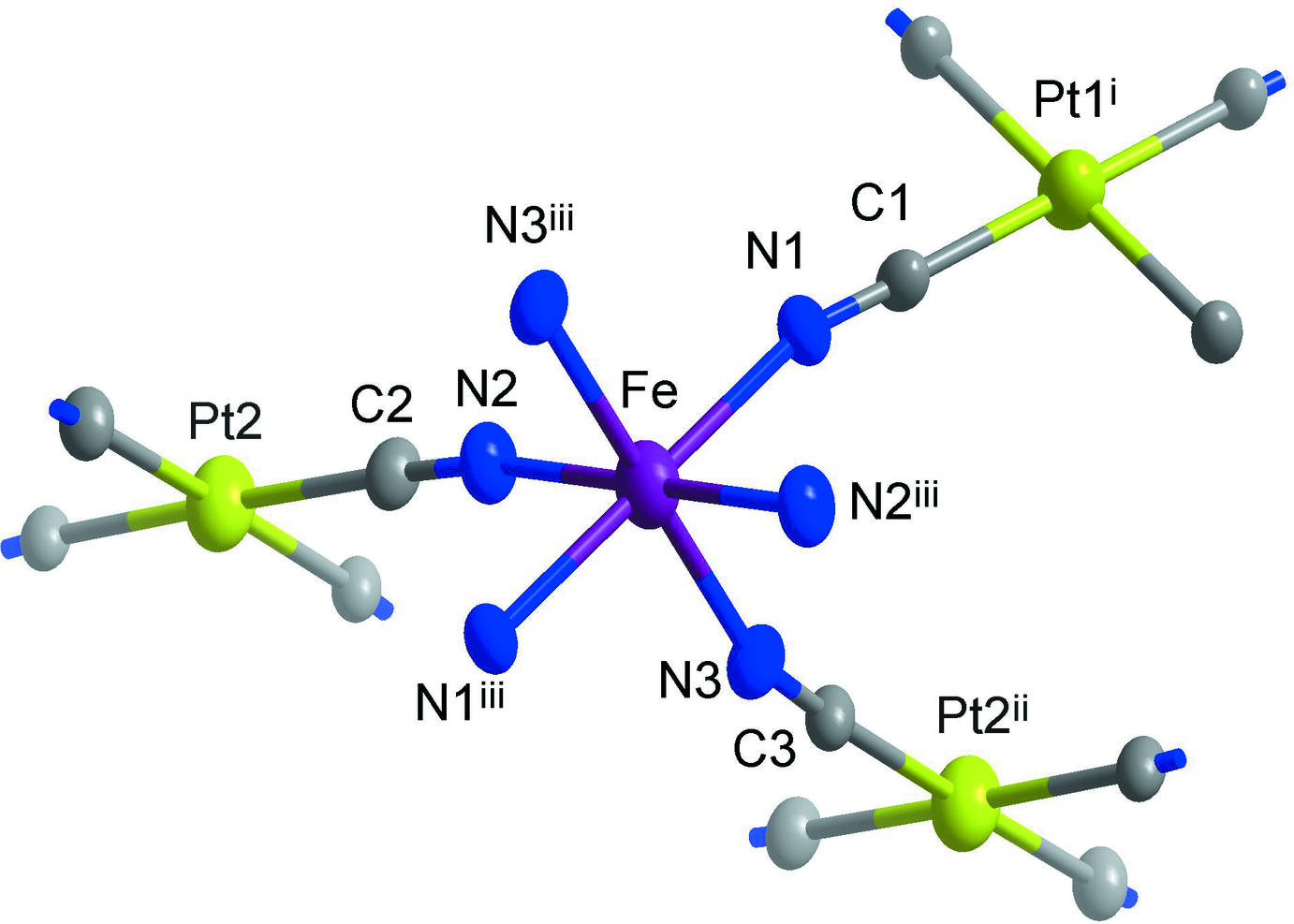

Supplement: Supplementary file 4 [file e-71-000i1-fig1.tif]

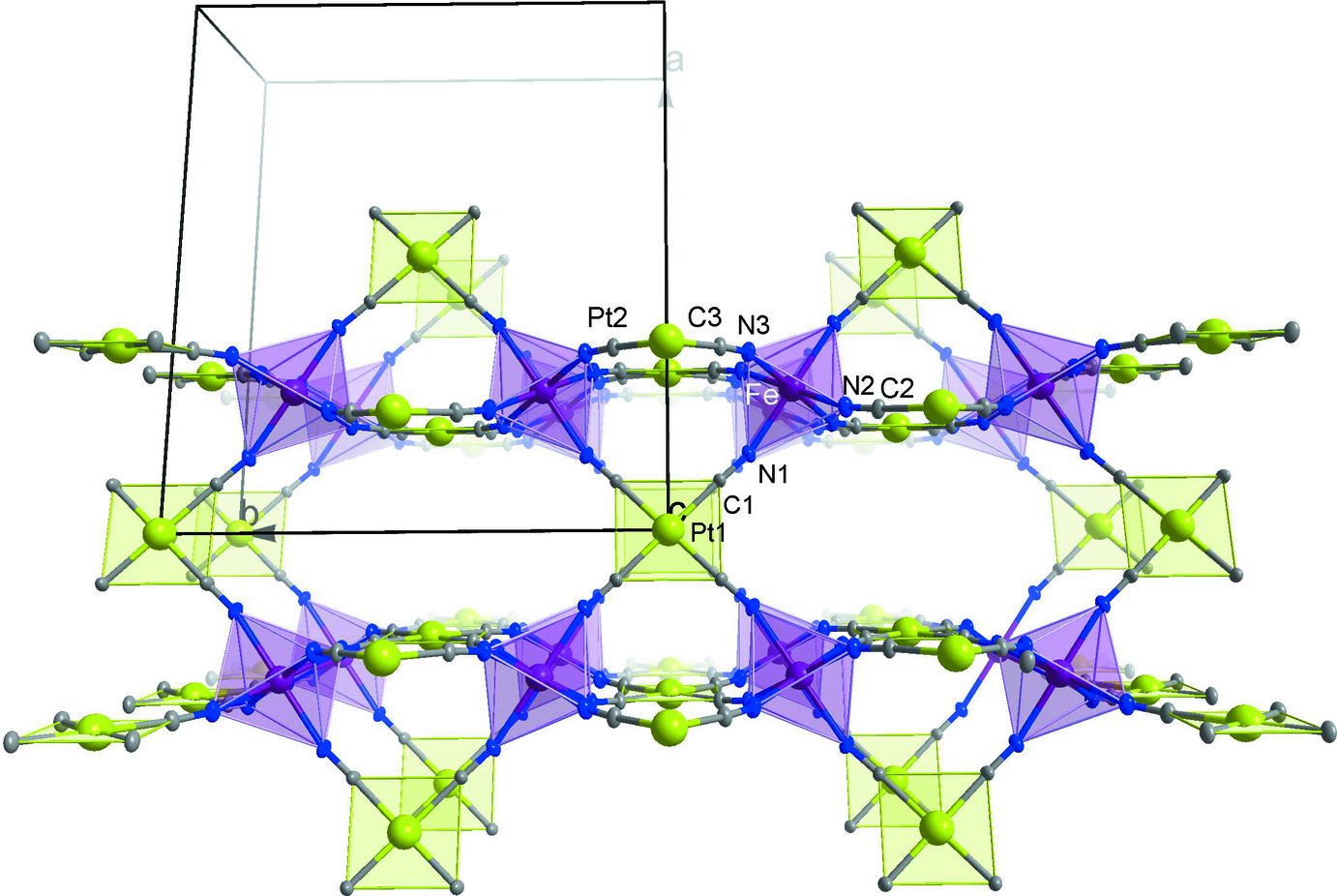

Supplement: Supplementary file 5 [file e-71-000i1-fig2.tif]
